# Assessing the Utility of ChatGPT Throughout the Entire Clinical Workflow: Development and Usability Study

**DOI:** 10.2196/48659

**Published:** 2023-08-22

**Authors:** Arya Rao, Michael Pang, John Kim, Meghana Kamineni, Winston Lie, Anoop K Prasad, Adam Landman, Keith Dreyer, Marc D Succi

**Affiliations:** 1 Medically Engineered Solutions in Healthcare Incubator Innovation in Operations Research Center (MESH IO) Massachusetts General Hospital Boston, MA United States; 2 Harvard Medical School Boston, MA United States; 3 Department of Radiology Massachusetts General Hospital Boston, MA United States; 4 Department of Radiology Brigham and Women's Hospital Boston, MA United States; 5 Data Science Office Mass General Brigham Boston, MA United States; 6 Mass General Brigham Innovation Mass General Brigham Boston, MA United States

**Keywords:** large language models, LLMs, artificial intelligence, AI, clinical decision support, clinical vignettes, ChatGPT, Generative Pre-trained Transformer, GPT, utility, development, usability, chatbot, accuracy, decision-making

## Abstract

**Background:**

Large language model (LLM)–based artificial intelligence chatbots direct the power of large training data sets toward successive, related tasks as opposed to single-ask tasks, for which artificial intelligence already achieves impressive performance. The capacity of LLMs to assist in the full scope of iterative clinical reasoning via successive prompting, in effect acting as artificial physicians, has not yet been evaluated.

**Objective:**

This study aimed to evaluate ChatGPT’s capacity for ongoing clinical decision support via its performance on standardized clinical vignettes.

**Methods:**

We inputted all 36 published clinical vignettes from the *Merck Sharpe & Dohme (MSD) Clinical Manual* into ChatGPT and compared its accuracy on differential diagnoses, diagnostic testing, final diagnosis, and management based on patient age, gender, and case acuity. Accuracy was measured by the proportion of correct responses to the questions posed within the clinical vignettes tested, as calculated by human scorers. We further conducted linear regression to assess the contributing factors toward ChatGPT’s performance on clinical tasks.

**Results:**

ChatGPT achieved an overall accuracy of 71.7% (95% CI 69.3%-74.1%) across all 36 clinical vignettes. The LLM demonstrated the highest performance in making a final diagnosis with an accuracy of 76.9% (95% CI 67.8%-86.1%) and the lowest performance in generating an initial differential diagnosis with an accuracy of 60.3% (95% CI 54.2%-66.6%). Compared to answering questions about general medical knowledge, ChatGPT demonstrated inferior performance on differential diagnosis (β=–15.8%; *P*<.001) and clinical management (β=–7.4%; *P*=.02) question types.

**Conclusions:**

ChatGPT achieves impressive accuracy in clinical decision-making, with increasing strength as it gains more clinical information at its disposal. In particular, ChatGPT demonstrates the greatest accuracy in tasks of final diagnosis as compared to initial diagnosis. Limitations include possible model hallucinations and the unclear composition of ChatGPT’s training data set.

## Introduction

Despite its relative infancy, artificial intelligence (AI) is transforming health care, with current uses including workflow triage, predictive models of utilization, labeling and interpretation of radiographic images, patient support via interactive chatbots, communication aids for non–English-speaking patients, and more [1-8]. Yet, all of these use cases are limited to a specific part of the clinical workflow and do not provide longitudinal patient or clinician support. An underexplored use of AI in medicine is predicting and synthesizing patient diagnoses, treatment plans, and outcomes. Until recently, AI models have lacked sufficient accuracy and power to engage meaningfully in the clinical decision-making space. However, the advent of large language models (LLMs), which are trained on large amounts of human-generated text such as those from the internet, has motivated further investigation into whether AI can serve as an adjunct in clinical decision-making throughout the entire clinical workflow, from triage to diagnosis to management. In this study, we assessed the performance of a novel LLM, ChatGPT (Open AI) [9], on comprehensive clinical vignettes (short, hypothetical patient cases used to test clinical knowledge and reasoning).

ChatGPT is a popular chatbot derivative of OpenAI’s Generative Pre-trained Transformer-3.5 (GPT-3.5), an autoregressive LLM released in 2022 [9]. Due to the chatbot’s widespread availability, a small but growing volume of preliminary studies have described ChatGPT’s performance on various professional exams (eg, medicine, law, business, and accounting) [10-14] and generating highly technical texts as found in biomedical literature [15]. Recently, there has been great interest in using the nascent but powerful chatbot for clinical decision support [16-20].

Given that LLMs such as ChatGPT have the ability to integrate large amounts of textual information to synthesize responses to human-generated prompts, we speculated that ChatGPT would be able to act as an on-the-ground copilot in clinical reasoning, making use of the wealth of information available during patient care from the electronic health record and other sources. We focused on comprehensive clinical vignettes as a model. Our study is the first to make use of ChatGPT’s ability to integrate information from the earlier portions of a conversation into downstream responses. Thus, this model lends itself well to the iterative nature of clinical medicine, in that the influx of new information requires constant updating of prior hypotheses. In this study, we tested the hypothesis that when provided with clinical vignettes, ChatGPT would be able to recommend diagnostic workup, decide the clinical management course, and ultimately make the diagnosis, thus working through the entire clinical encounter.

## Methods

### Study Design

**Figure 1:**
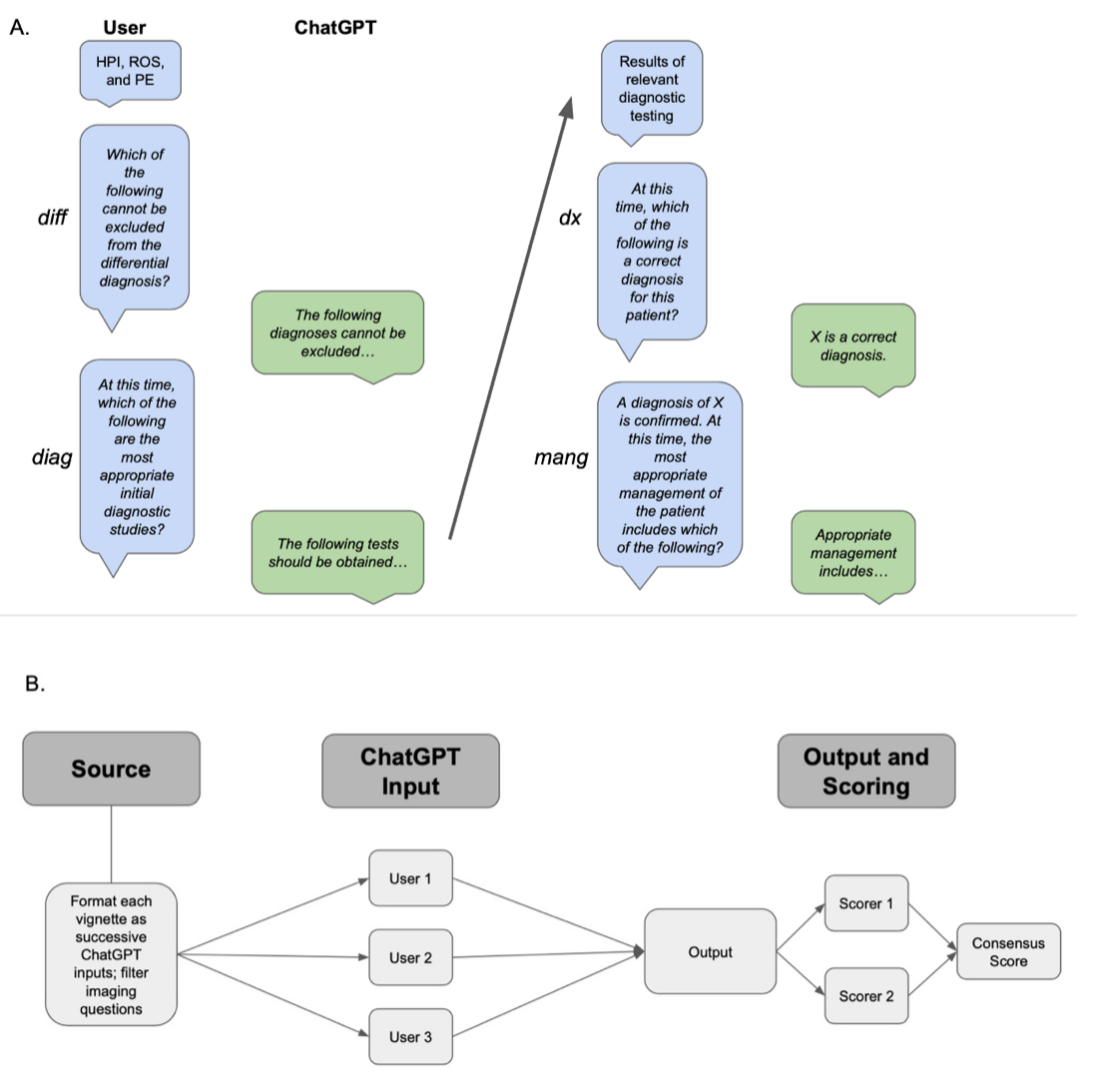
Experimental workflow for determining ChatGPT accuracy in solving clinical vignettes. Panel A: Schematic of user interface with ChatGPT for this experiment. Blue boxes indicate prompts given to ChatGPT and green boxes indicate ChatGPT responses. Nonitalicized text indicates information given to ChatGPT without a specific question attached. Panel B: Schematic of experimental workflow. Prompts were developed from Merck Sharpe & Dohme (MSD) vignettes and converted to ChatGPT-compatible text input. Questions requiring the interpretation of images were removed. Three independent users tested each prompt. Two independent scorers calculated scores for all outputs; these were compared to generate a consensus score. *diag*: diagnostic questions; *diff*: differential diagnoses; *dx*: diagnosis questions; HPI: history of present illness; *mang*: management questions; PE: physical exam; ROS: review of systems.

We assessed ChatGPT’s accuracy in solving comprehensive clinical vignettes, comparing across patient age, gender, and acuity of clinical presentation. We presented each portion of the clinical workflow as a successive prompt to the model (differential diagnosis, diagnostic testing, final diagnosis, and clinical management questions were presented one after the other; Figure 1A).

### Setting

ChatGPT (OpenAI) is a transformer-based language model with the ability to generate human-like text. It captures the context and relationship between words in input sequences through multiple layers of self-attention and feed-forward neural networks. The language model is trained on a variety of text including websites, articles, and books up until 2021. The ChatGPT model is self-contained in that it does not have the ability to search the internet when generating responses. Instead, it predicts the most likely “token” to succeed the previous one based on patterns in its training data. Therefore, it does not explicitly search through existing information, nor does it copy existing information. All ChatGPT model outputs were collected from the January 9, 2023, version of ChatGPT.

### Data Sources and Measurement

Clinical vignettes were selected from the *Merck Sharpe & Dohme (MSD) Clinical Manual*, also referred to as the MSD Manual [21]. These vignettes represent canonical cases that commonly present in health care settings and include components analogous to clinical encounter documentation such as the history of present illness (HPI), review of systems (ROS), physical exam (PE), and laboratory test results. The web-based vignette modules include sequential “select all that apply”–type questions to simulate differential diagnosis, diagnostic workup, and clinical management decisions. They are written by independent experts in the field and undergo a peer review process before being published. At the time of the study, 36 vignette modules were available on the web, and 34 of the 36 were available on the web as of ChatGPT’s September 2021 training data cutoff date. All 36 modules passed the eligibility criteria of having a primarily textual basis and were included in the ChatGPT model assessment.

Case transcripts were generated by copying MSD Manual vignettes directly into ChatGPT. Questions posed in the MSD Manual vignettes were presented as successive inputs to ChatGPT (Figure 1B). All questions requesting the clinician to analyze images were excluded from our study, as ChatGPT is a text-based AI without the ability to interpret visual information.

ChatGPT’s answers are informed by the context of the ongoing conversation. To avoid the influence of other vignettes’ answers on model output, a new ChatGPT session was instantiated for each vignette. A single session was maintained for each vignette and all associated questions, allowing ChatGPT to take all available vignette information into account as it proceeds to answer new questions. To account for response-by-response variation, each vignette was tested in triplicate, each time by a different user. Prompts were not modified from user to user.

We awarded points for each correct answer given by ChatGPT and noted the total number of correct decisions possible for each question. For example, for a question asking whether each diagnostic test on a list is appropriate for the patient presented, a point was awarded each time ChatGPT’s answer was concordant with the provided MSD Manual answer.

Two scorers independently calculated an individual score for each output by inputting ChatGPT responses directly into the MSD Manual modules to ensure consensus on all output scores; there were no scoring discrepancies. The final score for each prompt was calculated as an average of the 3 replicate scores. Based on the total possible number of correct decisions per question, we calculated a proportion of correct decisions for each question (“average proportion correct” refers to the average proportion across replicates). A schematic of the workflow is provided in Figure 1A.

### Participants and Variables

The MSD Manual vignettes feature hypothetical patients and include information on the age and gender of each patient. We used this information to assess the effect of age and gender on accuracy. To assess differential performance across the range of clinical acuity, the Emergency Severity Index (ESI) [22] was used to rate the acuity of the MDS Manual clinical vignettes. The ESI is a 5-level triage algorithm used to assign patient priority in the emergency department. Assessment is based on medical urgency and assesses the patient’s chief complaint, vital signs, and ability to ambulate. The ESI is an ordinal scale ranging from 1 to 5, corresponding to the highest to lowest acuity, respectively. For each vignette, we fed the HPI into ChatGPT to determine its ESI and cross-validated with human ESI scoring. All vignette metadata, including title, age, gender, ESI, and final diagnosis, can be found in Table S1 in Multimedia Appendix 1.

Questions posed by the MSD Manual vignettes fall into several categories: differential diagnoses (*diff*), which ask the user to determine which of several conditions cannot be eliminated from an initial differential; diagnostic questions (*diag*), which ask the user to determine appropriate diagnostic steps based on the current hypotheses and information; diagnosis questions (*dx*), which ask the user for a final diagnosis; management questions (*mang*), which ask the user to recommend appropriate clinical interventions; and miscellaneous questions (*misc*), which ask the user medical knowledge questions relevant to the vignette, but not necessarily specific to the patient at hand. We stratified results by question type and the demographic information previously described.

### Statistical Methods

Multivariable linear regression was performed using the *lm()* function with R (version 4.2.1; R Foundation for Statistical Computing) to assess the relationship between ChatGPT vignette performance, question type, demographic variables (age and gender), and clinical acuity (ESI). The outcome variable was the proportion of correct ChatGPT responses for each question and approximated a Gaussian distribution. Age and gender were provided in each vignette and are critical diagnostic information. Thus, they were included in the model based on their theoretical importance on model performance. ESI was included to assess the effect of clinical acuity on ChatGPT performance. Question type was dummy-variable encoded to assess the effect of each category independently. The *misc* question type was chosen as the reference variable, as these questions assess general knowledge and not necessarily active clinical reasoning.

## Results

### Overall Performance

**Figure 2:**
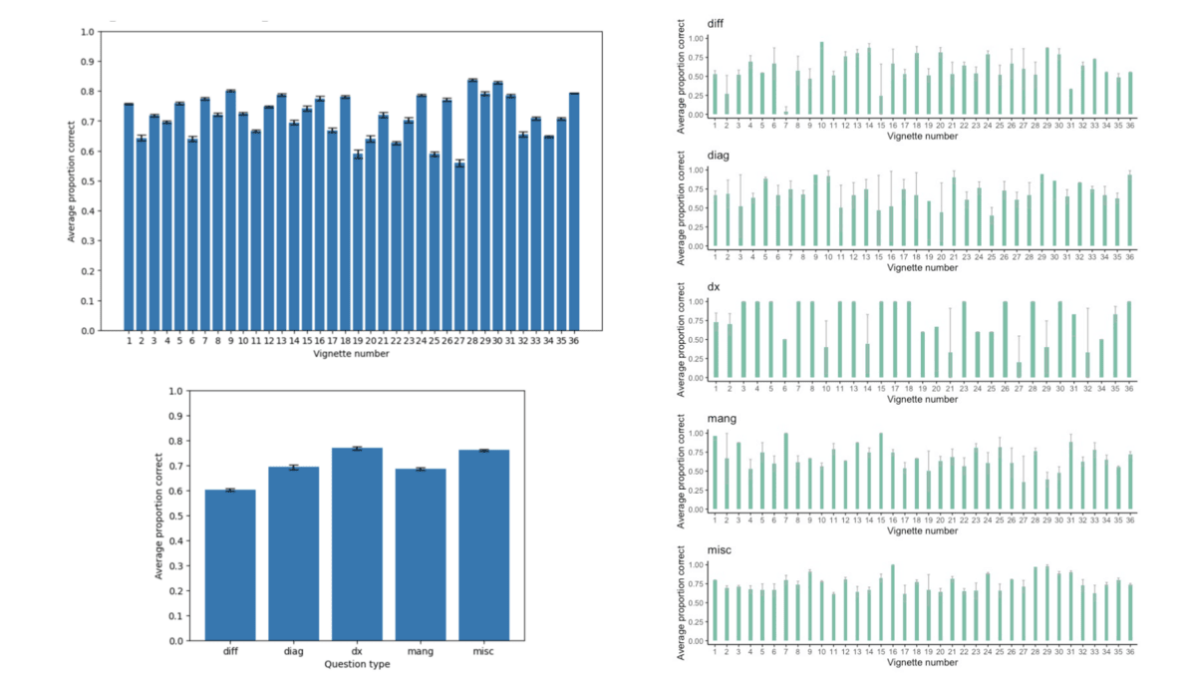
ChatGPT performance on clinical vignettes by vignette and question type. Panel A: ChatGPT overall performance for each of the 36 Merck Sharpe & Dohme (MSD) vignettes; error bars are 1 SE of the mean. Panel B: ChatGPT performance by question type; error bars are 1 SE of the mean. Panel C: ChatGPT performance by question type for each of the 36 MSD vignettes; error bars are 1 SE of the mean. *diag*: diagnostic questions; *diff*: differential diagnoses; *dx*: diagnosis questions; *mang*: management questions; *misc*: miscellaneous question.

Since questions from all vignettes fall into several distinct categories, we were able to assess performance not only on a vignette-by-vignette basis but also on a category-by-category basis. We found that on average, across all vignettes, ChatGPT achieved an accuracy of 71.8% (Figure 2A; Tables S2-S3 in Multimedia Appendix 1). Between categories and across all vignettes, ChatGPT achieved the highest accuracy (76.9%) for questions in the *dx* category and the lowest accuracy (60.3%) for questions in the *diff* category (Figure 2B; Table S3 in Multimedia Appendix 1). Trends for between–question type variation in accuracy for each vignette are shown in Figure 2C.

Vignette #28, featuring a right testicular mass in a 28-year-old man (final diagnosis of testicular cancer), showed the highest accuracy overall (83.8%). Vignette #27, featuring recurrent headaches in a 31-year-old woman (final diagnosis of pheochromocytoma), showed the lowest accuracy overall (55.9%; Figure 2A; Table S2 in Multimedia Appendix 1). These findings indicate a possible association between the prevalence of diagnosis and ChatGPT accuracy.

### Differential Versus Final Diagnosis

Both *diff* and *dx* questions ask the user to generate a broad differential diagnosis followed by a final diagnosis. The key difference between the 2 question types is that answers to *diff* questions rely solely on the HPI, ROS, and PE, whereas answers to *dx* questions incorporate results from relevant diagnostic testing and potentially additional clinical context. Therefore, a comparison between the 2 sheds light on whether ChatGPT’s utility in the clinical setting improves with the amount of accurate, patient-specific information it has access to.

We found a statistically significant difference in performance between these 2 question types overall (Figure 2B). Average performance on *diff* questions was 60.3%, and average performance on *dx* questions was 76.9%, indicating a 16.6% average increase in accuracy in diagnosis as more clinical context is provided. We also found that there were statistically significant differences in accuracy between *diff* and *dx* questions within vignettes for the majority of vignettes. This indicates that this is not an aggregate phenomenon but rather one that applies broadly, indicating the importance of more detailed prompts in determining ChatGPT accuracy, as *dx* prompt responses incorporate all prior chat session information and relevant clinical context (Figure 2C).

### Performance Across Patient Age and Gender

The MSD Manual vignettes specify both the age and gender of patients. We performed a multivariable linear regression analysis to investigate the effect of patient age and gender on ChatGPT accuracy. Regression coefficients for age and gender were both not significant (age: *P*=.35; gender: *P*=.59; Table 1). This result suggests that ChatGPT performance is equivalent across the range of ages in this study as well as in a binary definition of gender.

**Table 1:** Multivariable linear regression examining the relationship between ChatGPT accuracy and patient age, gender, and Emergency Severity Index (ESI), as well as question type.

Variable	Coefficient (%; 95% CI)	*P* value
Age	–0.05 (–0.17 to 0.60)	.35
Male gender	1.28 (–3.36 to 5.92)	.59
ESI	–0.98 (–4.15 to 2.96)	.55
*diag* ^a^	–6.62 (–13.42 to 0.18)	.06
*diff* ^b^	–15.80 (–22.90 to –8.70)	<.001
*dx* ^c^	–0.89 (–6.42 to 8.21)	.81
*mang* ^d^	–7.44 (–13.93 to –0.9)	.02

^a^*diag*: diagnostic questions.

^b^*diff*: differential diagnoses.

^c^*dx*: diagnosis questions.

^d^*mang*: management questions.

### ChatGPT Performance Across Question Types

*Diff* and *mang* question types were negatively associated with ChatGPT performance relative to the *misc* question type (β=–15.8%; *P*<.001; and β=–7.4%; *P*=.02, respectively). *Diag* questions trended toward decreased performance (*P*=.06); however, the effect was not statistically significant. There was no difference in performance in final diagnosis accuracy. The *R*^2^ value of the model was 0.083, indicating that only 8.3% of the variance in ChatGPT accuracy was explained by the model. This suggests that other factors, such as inherent model stochasticity, may play a role in explaining variation in ChatGPT performance.

### ChatGPT Performance Does Not Vary With the Acuity of Clinical Presentation

Case acuity was assessed by asking ChatGPT to provide the ESI for each vignette based only on the HPI. These ratings were validated for accuracy by human scorers. ESI was included as an independent variable in the multivariable linear regression shown in Table 1, but it was not a significant predictor of ChatGPT accuracy (*P*=.55).

### ChatGPT Performance Is Ambiguous With Respect to the Dosing of Medications

A small subset of *mang* and *misc* questions demanded that ChatGPT provide numerical answers, such as dosing for particular medications. Qualitative analysis of ChatGPT’s responses indicates that errors in this subset are predisposed toward incorrect dosing rather than incorrect medication (Table S4 in Multimedia Appendix 1). This may indicate that model training data are biased toward verbal as opposed to numerical accuracy; further investigation is needed to assess ChatGPT’s utility for dosing.

## Discussion

In this study, we present first-of-its-kind evidence assessing the potential use of novel AI tools throughout the entire clinical workflow, encompassing initial diagnostic workup, diagnosis, and clinical management. We provide the first analysis of ChatGPT’s iterative prompt functionality in the clinical setting, reflecting the constantly shifting nature of patient care by allowing upstream prompts and responses to affect downstream answers. We show that ChatGPT achieves an accuracy of 60.3% in determining differential diagnoses based on the HPI, PE, and ROS alone. With additional information, such as the results of relevant diagnostic testing, ChatGPT achieves an accuracy of 76.9% in narrowing toward a final diagnosis.

ChatGPT achieves an average performance of 71.8% across all vignettes and question types. Notably, of the patient-focused questions posed by each vignette, ChatGPT achieved the highest accuracy (76.9% on average) when answering *dx* questions, which prompted the model to provide a final diagnosis based on HPI, PE, ROS, diagnostic results, and any other pertinent clinical information. There was no statistical difference between *dx* accuracy and *misc* accuracy, indicating that ChatGPT performance on a specific clinical case, when provided with all possible relevant clinical information, approximates its accuracy in providing general medical facts.

Overall accuracy was lower for *diag* and *mang* questions than for *dx* questions (Figure 2B). In some cases, this was because ChatGPT recommended extra or unnecessary diagnostic testing or clinical intervention, respectively (Table S4 in Multimedia Appendix 1). In contrast, for several *diff* and *dx* questions (for which all necessary information was provided, as was the case for the *diag* and *mang* questions), ChatGPT refused to provide a diagnosis altogether (Table S4 in Multimedia Appendix 1). This indicates that ChatGPT is not always able to properly navigate clinical scenarios with a well-established standard of care (eg, a clear diagnosis based on a canonical presentation) and situations in which the course of action is more ambiguous (eg, ruling out unnecessary testing). The latter observation is in line with the observation from Rao et al [17], in that ChatGPT struggles to identify situations in which diagnostic testing is futile. Resource utilization was not explicitly tested in our study; further prompt engineering could be performed to evaluate ChatGPT’s ability to recommend the appropriate utilization of resources (eg, asking “What tests are appropriate clinically while also taking cost management into account?”).

Rao et al [17] found that for breast cancer and breast pain screening, ChatGPT’s accuracy in determining appropriate radiologic diagnostic workup varied with the severity of initial presentation. For breast cancer, there was a positive correlation between severity and accuracy, and for breast pain, there was a negative correlation [17]. Given that the data in this study cover 36 different clinical scenarios as opposed to trends within specific clinical conditions, we suspect that any association between the acuity of presentation and accuracy could be found on a within-case basis, as opposed to between cases.

Given the important ongoing discourse [3-8] surrounding bias in the clinical setting and bias in AI, we believe our analysis of ChatGPT’s performance based on the age and gender of patients represents an important touchpoint in both discussions [23-27]. Although we did not find that age or gender is a significant predictor of accuracy, we note that our vignettes represent classic presentations of disease and that atypical presentations may generate different biases. Further investigation into additional demographic variables and possible sources of systematic bias is warranted in future studies.

Although ChatGPT performs impressively on the surface, it is worth noting that even small errors in clinical judgment can result in adverse outcomes. ChatGPT’s answers are generated based on finding the next most likely “token”—a word or phrase to complete the ongoing answer; as such, ChatGPT lacks reasoning capacity. This is evidenced by instances in which ChatGPT recommends futile care or refuses to provide a diagnosis even when equipped with all the necessary information; this is further evidenced by its frequent errors in dosing. These limitations are inherent to the AI model itself and can be broadly divided into several categories, including misalignment and hallucination [28,29]. In this study, we identified and accounted for these limitations with replicate validation. These considerations are necessary when determining both the parameters of AI utilization in the clinical workflow and the regulations surrounding the approval of similar technologies in clinical settings.

An additional limitation of this study is the web-based availability of 34 of the 36 MSD Manual vignettes as of ChatGPT’s training data cutoff date. The contents of ChatGPT’s training data set are private; yet given that it was trained on large swaths of the internet, it is possible that the vignettes used in this study were also part of the training data set. However, since this study’s aims were to investigate the application of current tools in clinical decision-making, it is immaterial whether the vignettes were part of the training data set. The MSD Manual vignettes and answers represent the standard of care, making alignment between ChatGPT and vignette answers preferable in any context and the lack of alignment to be surprising.

As applications of AI grow more ubiquitous in every sector, it is important to not only understand if such tools are reliable in the clinical setting but also to postulate the most effective methods for deploying them. By analyzing ChatGPT’s accuracy at not just one step but rather throughout the entire clinical workflow, our study provides a realistic pilot of how LLMs such as ChatGPT might perform in the clinical settings. The integration of LLMs with existing electronic health records (with appropriate regulations) could facilitate improved patient outcomes and workflow efficiency. Our study provides important evaluation for the adoption of LLMs in clinical workflows and paves the way for future data-informed implementation.

## Appendix

Multimedia Appendix 1 Metadata for Merck Sharpe & Dohme (MSD) Manual vignettes, ChatGPT accuracy by vignette and question type, and ChatGPT raw output.

## Abbreviations

AI: artificial intelligence
*diag*: diagnostic questions
*diff*: differential diagnoses
*dx*: diagnosis questions
ESI: Emergency Severity Index
GPT-3.5: Generative Pre-trained Transformer-3.5
HPI: history of present illness
LLM: Large language model
*mang*: management questions
*misc*: miscellaneous questions
MSD: Merck Sharpe & Dohme
PE: physical exam
ROS: review of systems

## Data Availability

All data generated or analyzed during the study are included in the published paper.
